# Identification of thyroid hormone receptor binding sites in developing mouse cerebellum

**DOI:** 10.1186/1471-2164-14-341

**Published:** 2013-05-23

**Authors:** Remi Gagne, James R Green, Hongyan Dong, Mike G Wade, Carole L Yauk

**Affiliations:** 1 Environmental Health Science and Research Bureau, Healthy Environments and Consumer Safety Branch, Health Canada, Ottawa, ON K1A 0L2, Canada; 2 Department of Systems and Computer Engineering, Carleton University, Ottawa, ON, Canada

## Abstract

**Background:**

Thyroid hormones play an essential role in early vertebrate development as well as other key processes. One of its modes of action is to bind to the thyroid hormone receptor (TR) which, in turn, binds to thyroid response elements (TREs) in promoter regions of target genes. The sequence motif for TREs remains largely undefined as does the precise chromosomal location of the TR binding sites. A chromatin immunoprecipitation on microarray (ChIP-chip) experiment was conducted using mouse cerebellum post natal day (PND) 4 and PND15 for the thyroid hormone receptor (TR) beta 1 to map its binding sites on over 5000 gene promoter regions. We have performed a detailed computational analysis of these data.

**Results:**

By analysing a recent spike-in study, the optimal normalization and peak identification approaches were determined for our dataset. Application of these techniques led to the identification of 211 ChIP-chip peaks enriched for TR binding in cerebellum samples. ChIP-PCR validation of 25 peaks led to the identification of 16 true positive TREs. Following a detailed literature review to identify all known mouse TREs, a position weight matrix (PWM) was created representing the classic TRE sequence motif. Various classes of promoter regions were investigated for the presence of this PWM, including permuted sequences, randomly selected promoter sequences, and genes known to be regulated by TH. We found that while the occurrence of the TRE motif is strongly correlated with gene regulation by TH for some genes, other TH-regulated genes do not exhibit an increased density of TRE half-site motifs. Furthermore, we demonstrate that an increase in the rate of occurrence of the half-site motifs does not always indicate the specific location of the TRE within the promoter region. To account for the fact that TR often operates as a dimer, we introduce a novel dual-threshold PWM scanning approach for identifying TREs with a true positive rate of 0.73 and a false positive rate of 0.2. Application of this approach to ChIP-chip peak regions revealed the presence of 85 putative TREs suitable for further in vitro validation.

**Conclusions:**

This study further elucidates TRβ gene regulation in mouse cerebellum, with 211 promoter regions identified to bind to TR. While we have identified 85 putative TREs within these regions, future work will study other mechanisms of action that may mediate the remaining observed TR-binding activity.

## Background

Thyroid hormones (TH), triidothyronine (T3) and thyroxine (T4), are produced by the thyroid gland and are critically important for normal fetal and early neonatal development. T4 is a pro-hormone, which is converted to T3 by deiodinase enzymes [1]. T3 binds to various isoforms of thyroid hormone receptors (TRs) to initiate gene expression. TRs are encoded by two genes, *Thra* and *Thrb* that are expressed as TRα and TRβ. Each of these has alternative splice variants, i.e. TRα1, TRα2, THRβ1 and TRβ2 [2]. TR often binds in the presence of another TR or nuclear receptor (e.g. retinoid X receptor [RXR]) to form a stable DNA binding dimer or multimer [3]. Classic TH signalling involves interaction of T3 with TRs and co-regulators to control gene expression through interaction with DNA sequence elements known as thyroid response elements (TREs). Numerous factors can lead to perturbations in maternal TH levels, or impair the classic TH-TR-TRE response, and are associated with a suite of developmental defects including neurodevelopmental consequences. As such, various research efforts have focused on the identification and characterization of TH-TR-TRE interactions in order to understand the mechanistic processes associated with abnormal development under conditions of altered TH levels [4,5].

TREs can be divided into positive and negative TREs, which promote or inhibit gene transcription, respectively. TREs show considerable sequence variation and are therefore considered to be degenerate. Thus, precise characterization of the TRE sequence elements has been extremely difficult relative to more conserved motifs. In general, “classic” TREs are composed of 2 hexamers (often referred to as “half sites”) in direct repeat (DR), inverted repeat (IR) or everted repeat (ER) order. TR homodimers or heterodimers with RXR interact with the TRE hexamer motif. There are multiple factors, in addition to the consensus sequence, that determine whether a TR will bind to a DNA sequence. The orientation of hexamers and the number of nucleotides separating hexamer pairs in the response element is a discriminating factor for nuclear receptor binding to DNA [2]. For example, the dimer formed by the vitamin D receptor and RXR binds to 2 hexamers separated by a spacer of 3 nucleotides [6]. In contrast, published consensus TREs include non binding specific spacers of 4, 0 and 6 bps [7-9]. The current work describes the identification of putative TREs in genomic DNA through the development and application of computational analysis.

In an attempt to identify TREs involved in mitigating the neurotoxic effects of perturbations in thyroid hormone levels, our previous work screened TRβ-bound DNA prepared by chromatin immunoprecipitation from juvenile mouse cerebellum DNA using promoter microarrays (ChIP-chip; Agilent Technologies). This work examined ages bracketing a period of dynamic cerebellum development including an age immediately prior to (PND4) and at the peak of (PND15) a dramatic increase in circulating thyroid hormone levels in rodents (reviewed in Howdeshell [10]). In conducting this analysis, we used a blank subtraction followed by intra-array Lowess normalization, and peak detection using Chip Analytics 1.3 software [11]. Having originally applied the default analysis pipeline provided by the microarray vendor, in the present study we investigate more complex alternative computational approaches by leveraging recent quantitative benchmarking results and advances in the field of ChIP-chip data analysis.

In order to optimize methodological approaches for identifying TRE-containing DNA regions, we applied bioinformatics analyses to the ChIP-chip data acquired in our previous work [11]. We evaluated normalisation methods developed specifically for ChIP-chip data to compare their ability to correct biases that were present in our data. Next, we applied a computational method (Splitter [12]) using an approach evaluated by Johnson and colleagues [13] to identify genomic regions showing enrichment of TR binding. This peak finding algorithm models probe intensities with respect to their chromosomal location and was used to determine whether a binding event took place. Recent work by Johnson *et al.*[13] comprehensively evaluated spike-in ChIP-chip data produced from different platforms and relevant analytical approaches. Of these datasets, the Whitehead Institute dataset closely resembles our own experimental conditions since they also used the Agilent platform. The spike-ins used by Johnson *et al.* mimic sheared immunoprecipitated DNA with the advantage of knowing the precise chromosomal location and concentration. We used the spike-ins from the Whitehead Institute raw dataset to evaluate the sensitivity of peak identification using the normalization and peak finding analyses that we applied. In addition, we used the DNA sequence information associated with peaks to identify TRE hexamer characteristics and to conduct classic TRE mining. We then examined the frequency and distribution of TRE hexamers across known TH-responsive and non-responsive promoter regions, as well as random regions of the genome. Lastly, a number of predicted TR-binding regions were confirmed via ChIP-PCR. Our work describes a robust method for *in silico* prospecting for putative TREs for future confirmation, and enhances our understanding of the nature of TH regulation of gene expression.

## Results and discussion

### Normalization of ChIP-chip data

Euthyroid mice were sampled on post-natal days 4 or 15 and DNA from cerebella samples was subjected to ChIP using antibodies against TRβ. ChIP DNA was hybridized against total input DNA on Agilent custom gene promoter tiled DNA microarrays. Full ChIP-chip procedures are described in [11].

There are a number of sources of bias that may confound a microarray signal. For example, microarray probes with high G-C content – particularly if C/G residues occur at or near the probe ends – will tend to hybridize more effectively than probes with lower GC. We investigated methods of normalization to reduce the influences of these biases on microarray data.

A range of contemporary gene expression normalization methods were evaluated for their relevance to ChIP-chip data. Quantile [14] and Lowess [15] normalization methods, while widely adopted for gene expression datasets, were eliminated as viable options for ChIP-chip data normalization. The Quantile method is too stringent for probes whose spot intensities lie in the right tail of the intensity distribution [16]. Lowess normalization methods originally developed for 2-colour arrays [15] should be applied with great caution to ChIP-chip dataset because this normalization technique assumes symmetry along the average log intensity axis (A) of a log ratio versus log average intensity (MA) plot. ChIP-chip dataset typically do not show symmetry since binding events are characterized by probes exhibiting high positive log ratios, while large negative ratios are not expected to be observed.

Various normalization techniques have been developed specifically for ChIP-chip data. However, many of these are designed for single colour arrays. The method of Song *et al*. [17] was evaluated because it was developed for a two colour array. This method corrects for the GC bias that is induced at probe hybridization, however, it assumes a highly bimodal distribution of probe intensities. Unlike the strong bimodal distribution seen in their study linking the GC count and probe signal intensity fold change (see Figure three B of ref [17]), our data revealed a much weaker bimodal differentiation as illustrated in Additional file 1. We therefore did not pursue this normalization strategy.

**Figure 1:**
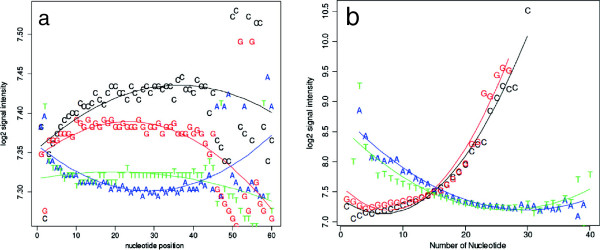
**Microarray signal intensity biases and post normalization plots with respect to nucleotide composition and count.** Both biases follow a polynomial trend that is unrelated to ChIP-chip signal. Since there is no relation between probe characteristics and signal, one should expect no trend in the data. **a**. The effect of base position along the probe on the signal intensity for the TI channel. A quadratic effect was observed for base position along the probe for A, C and G. **b**. Median log2 signal intensity of TI probes on the microarray versus the number of individual nucleotides in each probe.

A linear-based model was developed by Potter *et al*. [18] to correct multiple biases for two –colour chIP-methylation microarrays, which also uses immunoprecipitation. This model was used to determine the influence of probe characteristics as a source of bias in our data. Relative signal intensity in relation to nucleotide position and number was plotted as described in Potter *et al.*[18] (Figures two A and B). Figure 1a illustrates the median log2 signal intensities for one typical microarray for the total input (TI) sample. For each nucleotide, we plotted the median intensity of all probes that contain that nucleotide at each position within the probe. Quadratic regression was performed on the plot, and clearly showed that the data fit the quadratic model, particularly for the first 45 nucleotides of each probe, with the trend escaping correlation after the 45th position. Likewise, Figure 1b illustrates the relationship between nucleotide composition within the probe and signal intensity. Again, it is apparent that the relationship follows a quadratic curve. Therefore, a slightly modified version of Potter’s method of normalization (in which an additional quadratic term relating nucleotide composition and nucleotide position was used) was examined further below, in the context of peak identification.

### Peak Identification for ChIP-chip data

Johnson *et al.*[13] found the Splitter method [18] to be the optimal approach for peak identification for Agilent tiling arrays (based on a spike-in experiment). Thus, this approach was applied to our dataset. Table 1 summarizes the key experimental parameters for our study and for the Whitehead Institute dataset. The Whitehead dataset was comprised of two replicate microarrays hybridized with a spike-in mixture. The spike-in mixture holds 100 randomly selected known human genomics DNA sequences (targets) in predicted promoter regions [19]. Each target was prepared at a concentration of 500 pg/μL and then diluted individually 1.25 fold to 196 fold to create ladders of enrichment levels of each target. The targets were then added to a commercial human genomic DNA preparation (see [13] for details). The resulting spike-in solution was sent to volunteer labs that analyzed blindly the sample and returned lists of peaks to the coordinator or the experiment. We first re-analyzed the Whitehead Institute dataset to determine the influence of normalization on the sensitivity and precision of peak identification using Splitter, since this was not discussed in [13]. Furthermore, the specific range of probe intensities considered as potentially forming peaks (i.e. the “from” and “to” parameters of Splitter [12]) was not specified and the authors indicate that they estimated these parameters based on visual inspection of the probe intensity histograms. As such, direct experimental replication was not possible. Therefore, we re-evaluated the Johnson *et al.* data using a systematically defined probe intensity cut-off to find the optimal probe intensity threshold values. These optimized parameters were later applied to our own data.

**Table 1:** Media characteristics comparison between Whitehead and Dong

	**Johnson *et al*.**	**Dong *et al.***
Probe length (nucleotides)	60	60
Median genomic region between probes (nucleotides)	10	153
Number of probes/array	244 000	44 000
Average length of DNA segment hybridized to the array	497	600
Amplification technique	Ligation-Mediated Amplification	Whole Genome Amplification

**Figure 2:**
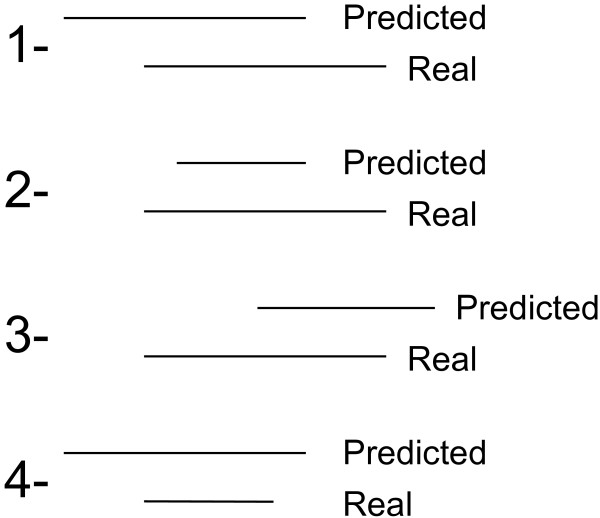
**True positive binding site calls.** Illustration of the 4 cases when a predicted binding site is called as a true positive. This figure shows the possible cases of overlap between a predicted peak and true location of a peak.

To optimize the Splitter intensity threshold parameter for the Agilent platform, we conducted a series of analyses over a range of log ratio cut-off intensities from 2.25 to 2.75 standard deviations of the distribution, with and without prior application of normalization, to the Whitehead dataset. Normalization was also applied to each individual channel prior to peak finding. However, no improvement in peak recovery accuracy was observed. Accuracy was measured in terms of true positives (TP), false positives (FP), and false negatives (FN). Here, a predicted peak is identified as a TP if a predicted binding site overlaps with the true starting or ending location of a spike-in DNA segment (see Figure 2). A predicted binding site that doesn’t fall within these 4 cases is labelled as a FP, while a missed spike-in DNA segment constitutes a FN.

**Figure 3:**
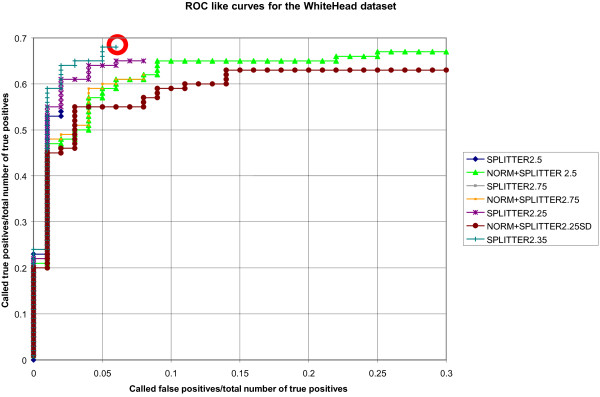
**True positive hit rates vs. false positives calling rates for the Johnson et al.** Dataset for Combinations of Normalization and Peak Finding. Several curves are plotted for both normalized (curves labelled with NORM) and non-normalized data. The various Splitter standard deviations are also noted for each curve (e.g., Splitter2.35 indicates SD set to 2.35). Please note, the curve for NORM+Splitter2.35 completely overlaps with Splitter2.35, and thus was not plotted (for clarity). The highest TP/FP ratio corresponds to the data point circled in red. This represents the optimum standard deviation cut-off parameter value for Splitter.

We have plotted performance curves as in Johnson *et al*. (analogous to receiver operating characteristics curves), which plot (called TP) / (total number of TP) versus (called FP) / (total number of TP) using the Whitehead dataset [13]. Figure 3 illustrates the performance curves for Splitter with and without normalization over a range of probe intensity threshold values (see Methods). In order to maximize the TP rate while simultaneously minimizing the FP rate, one should select the curve that is the closest to the upper-left corner of the graph (indicated by a circle in Figure 3). In this case, the best cut-off value for Splitter [12] was found to be 2.35 SD with no normalization. Despite the overlap of Splitter 2.35 SD with and without normalization, the remaining models that applied normalization showed consistently lower sensitivity relative to non-normalized data. Thus, the non-normalized data, with SD 2.35 were selected for subsequent analyses.

**Figure 4:**
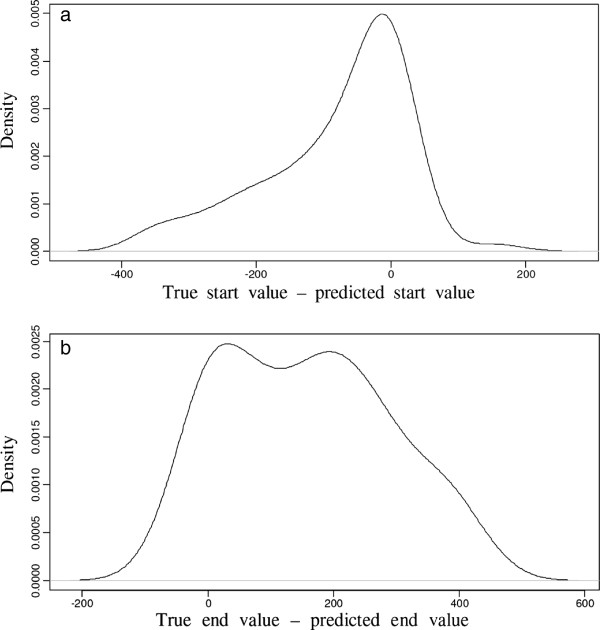
**Differences between peak width true values and splitter predicted values for the Whitehead dataset. a**. Difference between the true and predicted start values for the true positives in the Whitehead dataset. **b**. Difference between the true and predicted end values for the true positives in the Whitehead dataset. On the y-axis, density is the empirical estimate of the underlying probability density function.

For each predicted peak, Splitter outputs a chromosome number, a starting location, and an ending location. Using the optimal threshold value of the Whitehead dataset, 68 out of 100 true peaks were successfully predicted by Splitter. For each predicted peak, the difference between the predicted and actual start and end of the genome location were calculated to check for systematic over- or under-estimation of peak width. Since a true positive was labelled when any overlap occurred between true and predicted peak regions (Figure 2), histograms were generated for the differences in predicted and true DNA segment extents. Figures 4a&b show the positional biases from the peak start and end prediction, calculated as (true start genome location - predicted start genome address) and (true end genome address - predicted end genome address) respectively. These histograms reveal a strong bias towards under-predicting peak extents, particularly for the estimated peak 3’ end. With these findings in mind, all peaks predicted by Splitter in our own dataset were corrected by adding 200 bp to the 3’ end, widening the peak.

The last two Splitter parameters to establish for the analysis are MINRUN and GAPMAX. MINRUN specifies the minimum number of adjacent probes that must have intensities above threshold to constitute a “peak”. This value will depend on the density/tiling of the probes across the promoter region and the expected length of DNA fragments following sonication. MINRUN must be set to at least two in order to minimize false positives. In contrast, setting the MINRUN to 3 may be overly conservative and lead to too many missed positives. Using the Keles *et al.*[20] equation, the number of probes expected per peak is 3.09 for the Agilent arrays used in this study, where the mean length of shredded DNA is expected to be 600 bp. However, Keles *et al.* note that the distribution of the length of shredded DNA is left shifted, leading to the expectation that many true hits will have fewer than three probes per peak. Therefore, we have set the MINRUN parameter to 2 in this study.

The GAPMAX parameter is the longest unmapped genomic section permitted between two probes while still considering the two probes to be ‘adjacent’. In the Johnson *et al.* study, the GAPMAX applied by the Whitehead Institute was set to 200 to cover the mapping of 3 probes (60 bp each) plus 2 gaps (10 bp each). Since probe mapping intervals are variable in length (e.g. small repeat regions are not covered by probes on the array) it is prudent to extend GAPMAX to allow for one omitted probe. Using our own media characteristics (Table 1), this strategy would require a GAPMAX of 488 bp for our own TR data. It is important to note that the Whitehead dataset was performed with a spike-in sample, which is expected to be less noisy than a real sample. Thus, the results in Additional files 2 and 3 are generated using a Splitter GAPMAX parameter value of 488, but priority for the evaluation of the results should be given to peaks where mapping with lower inter-probe intervals (see Gapmax column of Additional files 2 and 3).

### Analysis of TR ChIP-chip data

Following the findings above, the TR ChIP-chip dataset of Dong *et al.* was analyzed without normalization using Splitter peak finding with a cut-off value of 2.35, a MINRUN of 2, and GAPMAX of 488. As mentioned above, Splitter tends to systematically underestimate the length of peaks (Figure 4). Therefore, the 3’ end of each peak was corrected by adding 200 bp. The list of identified peaks, or TR-enriched DNA segments, found by applying Splitter to our ChIP-chip data is found in Additional files 2 and 3. These tables contain information such as the nearest gene and position of TRE with respect to the nearest gene. Briefly, 44 peaks were identified for euthyroid mice collected on post-natal day 4 (PND4) and 186 for PND15, for a total of 230 candidate binding sites. Of these, 19 were found to overlap between PND 4 and 15 (see Additional file 4), leading to 211 unique candidate binding sites. Twenty-five peaks were subjected to experimental validation through ChIP-PCR, of which 16 were identified as enriched over background by ChIP-PCR and thus called ‘positive’ (i.e., TP). Nine peaks were not enriched by ChIP-PCR and thus were considered to be FP. Although ChIP-qPCR has its own source of errors, these errors are not expected to correlate systematically, so there is value in applying both methods (ChIP-chip & Chip-PCR) as a means of validation. This outcome is included in the “Validation” column of the tables. A highly conservative approach was used, where only the sub-sequence immediately surrounding the predicted peak was tested for TR binding. Therefore, we are highly confident in all 16 true positive sites. Validated peaks are used in later sections as true positives and false positives for finding TREs. A comparison of the binding sites found in the present analysis to the ones found in Dong et al. [11] revealed a total of seven PND15 peaks in common in both analyses (see Additional file 4 for details).

### Presence of TRE Hexamers in genomic DNA

**Figure 5:**
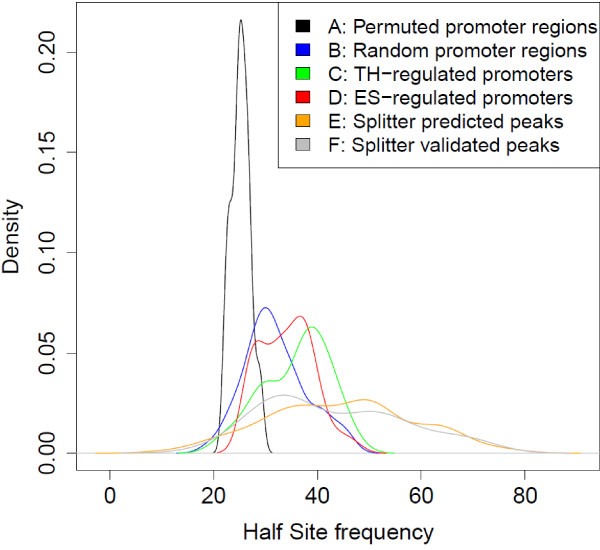
**Density of TRE consensus Hexamers for various promoter groups.** Sequences in promoter groups a-f were scanned for half sites with a PWM [40] score threshold of 6.0. Probability density functions for the number of TRE half sites observed per 1000 bps are shown for each class of DNA sequence. Estrogen regulated genes (group d) were chosen for this comparison since the characterized motif for their nuclear receptor is highly similar with the exception that the orientation of the motif is a palindrome with a 3 nucleotide spacer. These curves show the density of TRE half sites per 1000 bps for: **a**. in permutations of random promoter regions (n = 50). **b**. Randomly selected promoter regions (n = 50). **c**. promoter region for genes with TH regulated promoter regions (n =28). **d**. promoter region for genes with estrogen regulated promoter regions (n = 50). **e**. Regions detected by Splitter (n = 100), and **f**. validated regions detected by Splitter (n = 36). The results shown by a are random nucleotide permutations of the promoter regions shown in **b**. On the y-axis, density is the empirical estimate of the underlying probability density function. Regions **a**-**d** are 10 kbps in length where regions **e**,**f** are in the 300-600 bps range.

As described above, TREs are composed of 2 hexamers or half sites. We investigated whether the density of hexamers could help identify genomic regions containing TREs. We hypothesised that a higher density of hexamers in a genomic region would indicate a higher probability of finding a TRE. A position weight matrix (PWM) model was built using all previously reported half sites (Table 2) to represent the classic TRE hexamer sequence motif. Briefly, a PWM compiles information content (log odd values) for each nucleotide position using existing information on known binding sequences for the protein of interest. Scores for DNA sequences of interest can then be calculated by respectively summing the PWM matrix values for each position. The presence and abundance of TRE hexamers was determined for various different types of genomic DNA sequences. The DNA regions examined included: (A) permutations of random promoter regions (-8 kb to +2 kb of transcription start site); (B) randomly selected promoter regions (without permutation); (C) promoter regions for genes that are known to be regulated by TH (see Methods); (D) promoter regions for genes that are regulated by estrogen; (E) regions corresponding to the peaks detected by Splitter; and (F) Splitter detected regions validated through ChIP-PCR to be truly TR-binding. Estrogen regulated genes were included in this comparison since the characterized motif for this nuclear receptor is highly similar to TREs, with the exception that the orientation of the motif is a palindrome with a 3 nucleotide spacer [21]. Figure 5 shows the distribution of TRE hexamer frequency (per Kbp) within each class of promoter sequence. The analysis revealed that permuted promoter regions contain significantly (p < 0.0001) fewer half sites than non-permutated promoter regions (Figure 5a vs. b). The data suggest a nucleotide position importance for promoter sequence composition, since permutations of identical sequences show a much lower number of hexamers. Interestingly, the promoters of established TH-regulated and estrogen-regulated genes (Figure 5 groups c & d) appear to exhibit bi-modal distributions, where one mode behaves similarly to the random promoters (Figure 5 group b) and another mode has half-site frequencies well above what one would expect by chance. We therefore conclude that, while the occurrence of the TRE motif is strongly correlated with gene regulation by TH for some genes, other TH-regulated genes do not exhibit an increased density of TRE half-site motifs. Figure 5 (groups e & f) shows the distribution of half sites in Splitter predicted and Splitter validated regions. The distribution of TRE motifs across these regions is broader and half site frequency is much higher than for randomly selected promoter regions (p < 0.0001). The broader distribution of these regions indicates higher variability in the number of hexamers for these classes of sequence. It is interesting to note that the Splitter peak regions are also significantly enriched for TRE motifs when compared to the promoter regions of previously reported TH-regulated genes (p < 0.003). However, it should be noted that these Splitter peak regions are much smaller (300-600 bp) than the 10 kb scanned for the other promoter sequence classes examined, which may lead to an apparent concentration of TRE half sites. That is, the concentration of TRE half sites may be ‘diluted’ when examining an entire promoter region vs. the subsequence that ChIP-chip indicates is TR-binding.

**Table 2:** List of TRES found in the literature

**Gene**	**Accession #**	**GS**	**Location**	**TS**	**Sequence**	**Type**	**Ref.**	**TRE config.**
Nr4a1	NM_010444	+	-1182 to -1218	+	actgggatggagatgtgacctgcagggtga	Neg	[22]	Trimer
c-myc	NM_019660	+	Exon1	+	cgacctaagaaggcagctct	Neg	[23]	Dimer
KLF9	NM_010638	+	-3830 to -3804	+	aggtgaagtgaggtca	Pos	[24]	DR4
UCP3	NM_009464	+	-59 to -30	+	tcagaattaggtttcaggtcagctggtgca	Pos	[25]	DR1
Myogenin	NM_031189	+	-526 to-494	+	gtggtaggtctttaggggtctca	Pos	[26]	DR4
Nas1	NM_019481	-	-436 to -425	-	aggctatagccc	Pos	[27]	IR0
MLXIPL	NM_021455	+	-2436 to-2389	+	cgggtactagagggca	Pos	[28]	DR4
MLXIPL	NM_021455	+	-2436 to-2389	+	aggcaatgagaggtga	Pos	[28]	DR4
ABCD2	NM_011994	-	-401	-	tggcctgattcgacct	Pos	[29]	DR4
CYP7A1	NM_007824	-	-3KB	-	agggca	Pos	[30]	Monomer
CYP7A1	NM_007824	-	-3KB	-	aggtcagggtca	Pos	[30]	DR0
Fgfr1	NM_010206	+	-279 to-264	+	ttgcccatttcaacct	Pos	[31]	DR4
MBP	NM_001025251	+	-184 to-167	+	acctcggctgaggaca	Pos	[9]	DR4
TRH	NM_009426	-	-57 to-51	-	tgacct	Neg	[32]	Monomer
(RAT) MBP	NM_001025289	+	-186 to-163	+	agacctcggctgaggaca	Pos	[33]	ER6

List of documented TREs in mouse genome compiled from the literature. Column description goes as follow; the gene that is regulated by the TRE, its accession number, its DNA strand (GS), the location with respect to the transcription start site of the gene, the strand of the TRE (TS), the sequence which contains the TRE with binding site in bold, the type of gene regulation that the TRE performs (up or down regulates gene transcription), the literature reference for the TRE and the TRE configuration are shown.

**Figure 6:**
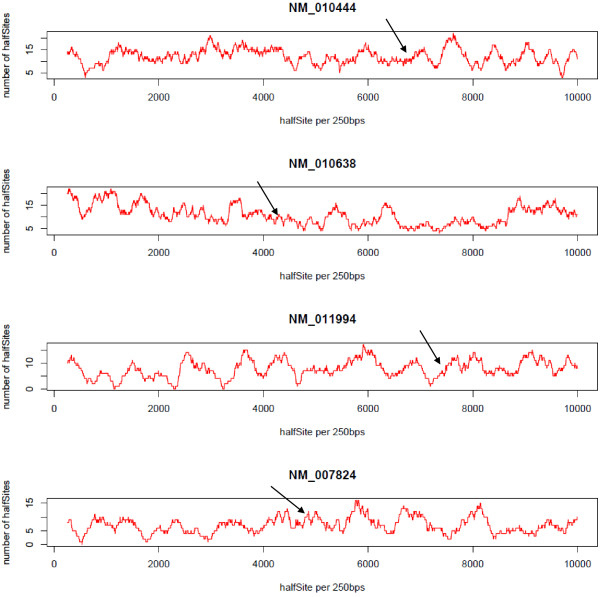
**Density of TRE half sites in promoter regions of 4 TH regulated genes.** Each graph shows the NCBI Reference sequence ID of the T3 regulated gene and its chromosomal location. The Y axes indicate the number of half sites and the X axis indicates the nucleotide position with respect to the gene, where 0 = -8 kbps from the transcription start site. The arrows show precisely where the TREs are located for each known TH regulated gene.

The above findings led us to examine the promoter regions of the published genes that are known to be regulated by TH in more detail and to determine if the sub-sequence most likely to be TR-binding can be determined by examining half site frequency. A sliding window of 250 bp was used to examine the number of half sites within the promoter regions of TH-regulated genes from Table 2. We have used a stringent procedure to identify the actual TRE location from the literature, resulting in only 4 TH-regulated genes for which the actual TREs could be unambiguously determined. Figure 6 plots the half site density (y-axis) along the length of the promoter region sequence (x-axis) with the actual TRE location highlighted using an arrow. This analysis revealed that there is no significant increase in the density of half sites near the actual TRE compared with the remainder of the promoter region. This analysis was repeated on the remaining eight mouse TH-regulated genes from Table 2 (see Additional file 5); however, it should be noted that the true TRE location for many of these genes could not be recovered with high confidence due to the constant revision of the mouse reference genome and ambiguities in the original literature. Consistent with our findings in Figure 6, all eight genes exhibit a lack of correlation between half-site frequency and TRE location. We therefore, conclude that half site frequency and density do not appear to be predictive of TRE location within the promoter region of some known TH-regulated genes and we were unable to use these features to garner confidence in true positive peaks identified using ChIP-chip.

### Classic TRE Identification

**Figure 7:**
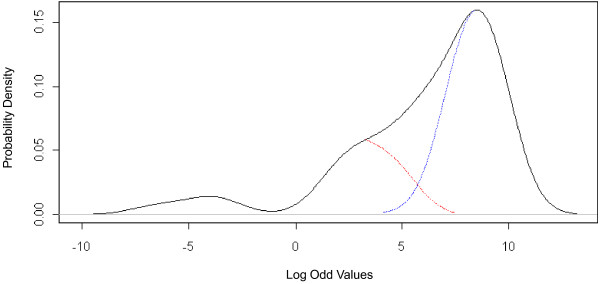
**Histogram of PWM scores from half sites from the literature.** {DR4, IR0, ER6} TREs gathered from the literature were split into the two hexamers and aligned to create a list of half sites. PWM construction and scoring was applied to the known half sites using a leave-one-out (LOO) test protocol. This resulted in a left skewed score distribution. The distribution can be broken into 2 sections, a right region (blue) with high scores and a small left region (red). The negative scores are driven by half sites containing two C’s in positions 2 & 3 instead of G‘s. The Y-axis of Figure 7 reflects the probability density of observing a particular log-odds value from the PWM model for half-sites.

By using a leave-one-out (LOO) protocol over all half sites from the known TREs in Table 2, we are able to examine the degree to which each half site of each TRE matches the classic TRE motif. In the LOO protocol, a single half site from one TRE is withheld, while the remaining known half sites are used to create a PWM. The withheld half site is then compared against the PWM model and its score is recorded. This allowed a non-biased PWM score to be computed for each half site since that half site was not used to compile the matrix. The resulting left skewed PWM score distribution is shown in Figure 7. One hypothesis for the left shifted distribution in Figure 7 is that TRE dimers are primarily composed of one low scoring half site and one high scoring half sites. The difference in score threshold between half sites may be explained by the heterogeneity of some TR binding complexes (e.g. TR-RXR). Bootstrapping experiments were conducted on both known mouse TRE dimers and also known TREs from other species described in Williams and Brent [34]. Given our sample size, there was insufficient evidence to reject the null hypothesis that both scores in each pair of half sites were, in fact, drawn from the same distribution (see Methods). This hypothesis should be further explored as more TREs are confirmed in the literature. However, visually, a bimodal distribution does appear to explain the left shifted distribution seen in Figure 7. Here the two possible component distributions are shown using blue (high scores) and red (lower scores). The negative scores are driven by half sites containing two C’s in positions 2 & 3 instead of G’s present in the canonical consensus half site.

Based on the results above, we propose a new dual-threshold model for identifying potential TREs. For a given putative TRE site, the score of each half site should be computed. A putative TRE should be composed of one half site whose PWM score exceeds a lower threshold, t_1_, and the one half site with a PWM score exceeding a higher threshold, t_2_. If the two scores comply with this rule, the putative site has a high probability of being a true TRE. Approaches have been developed in the literature to find bi partie binding sites [35-37]. These approaches find symmetric highly conserved half sites spaced by non conserved nucleotides. Here, we use TREs found in the literature and take advantage of the non-unimodal distribution of half sites to maximize sensitivity and specificity.

A cross-validation experiment was conducted to optimize the two threshold values in order to minimize the false positive rate while maximizing the sensitivity. The test dataset was composed of sequences taken from the genomic regions surrounding previously reported TREs found in the literature (Table 2). For the cross-validation experiment, known TREs that did not correspond to consensus motifs (DR4, IR0, ER6) were excluded in order to simplify the analysis. Also, due to the various genome builds available, some TRE sequences could not be found within their respective genomes and were also excluded. Since the median length of the ChIP-chip sequences in the present study is 520 bases, we used test sequences of this length, centred on the known TRE. In addition, false positives were also found in our ChIP-chip dataset through experimental validation in the wet-lab (Additional files 2 and 3, “Validation” column). These were used as true negative sequences in the cross-validation analysis. In total, our cross validation dataset was composed of 10 TP sequences and 9 TN sequences.

The LOO cross validation experiment was conducted by evaluating our dual-threshold PWM scanning model using every possible pair of threshold values (where score is measured as the sum of log odd values) over the ranges t_1_ = {3, 3.05, 3.1…..5} and t_2_ = {5, 5.05, 5.1….8} to identify TREs in the 19 sequences described above. The optimal parameter values found by the LOO cross validation were t_1_ =7 3.76 and t_2_ = 6.0, obtaining a true positive rate of 0.73 and a false positive rate of 0.2.

To further validate our dual-threshold PWM method, we attempted to identify the known mouse TREs in the sequences included in Table 2. Of these 16 mouse sequences known to be TH-regulated, only seven of them had the form DR4, IR0, or ER6 (the forms that our dual-threshold model searches for). Of these seven, we correctly predicted four of them (NM_010638, NM_031189, NM_021455 site 2, NM_011994) and missed three (NM_019481, NM_021455 site 1, NM_001025251). Of the three missed TH-regulated genes, NM_001025251 has a non-canonical half site (acctcg) which would not be found by our model (the ‘cc’ at positions 2-3 results in a negative half-site score as discussed above). This leads to an overall TPR of 0.6 which, given the small sample size (n = 7), effectively agrees with our expected TPR as measured by the LOO results above.

Once the optimal parameter values were determined, our novel dual-threshold PWM scanning model was applied to the two sets (PND4 & PND15) of TRE ChIP-chip data to scan for putative TREs. Additional files 6 and 7 present lists of TRE candidates found from the TR-binding regions identified by Splitter from PND4 and PND15 mice respectively. We identified 15 potential TREs within 44 Splitter peaks for PND4 and 80 potential TREs in 186 Splitter peaks for PND15. Ten out of the 15 TREs identified in the PND4 samples were also found in the PND15 samples, providing validating support that they are true TREs operating across various developmental time points. Of the potential TREs (DR4, IR0, ER6), three in PND4 and ten in PND15 show multiple TREs within a single Splitter identified ChIP-chip region. In other words, more than one TRE consensus was found within a single DNA region corresponding to a Splitter region. This interesting phenomenon may be explained by the tendency for transcription factor binding sites, in general, to appear in groups where one or more could be functional in gene regulation. These additional TF binding sites are often referred to as “shadows” [38].

We then applied our dual-threshold PWM method to all six classes of sequences used to plot half-site frequency in Figure 5. An analogous figure, showing a smoothed histogram of the number of sequences exhibiting a given dual-threshold model score for each class of sequences is now included in Additional file 8. While the permuted and randomly selected promoter regions tend to have uniformly low scores (as expected), it is interesting to note that TH-regulated genes, Splitter regions, and Validated regions all exhibit a clear bimodal distribution. One sub-class of these sequences appears to score much higher than would be expected by chance, while another sub-class of sequences does not. This could perhaps be explained by two different mechanisms of TH-regulation: one involving direct binding of TR to DNA and therefore exhibiting strong TRE motifs, and one involving indirect binding of TH to DNA, perhaps through an intermediary transcription factor as suggested by Lazar [39] or perhaps through an as-yet uncharacterized process.

## Conclusions

We used ChIP-chip normalization techniques to evaluate a slightly modified version of Potter *et al.*[18]*,* for correcting for nucleotide composition and position within the probe. Although normalization appears to correct for bias when applied in isolation, it was found to not be beneficial when combined with the Splitter peak finding algorithm. The Splitter algorithm for peak finding was modified to our dataset, and revealed 230 high confidence predicted peaks in PND4 and PND15. Of 25 peaks selected for ChIP-PCR validation, 16 peaks were determined to be truly TR-binding.

We tested the hypothesis that half site density within a promoter may be indicative of TH-gene regulation. Randomly selected promoter regions had a significantly higher density of half site sequence motifs than permutated sequences. However, genes known to be regulated by TH and ES appear to show a bimodal distribution, where one set of sequences appears to be enriched with half sites, whereas the other set does not exhibit significant enrichment. The fact that many TH-regulated promoter sequences had significantly fewer half sites than Splitter predicted peak regions may be explained by the fact that TRE motifs may only be enriched near the actual TR-binding site, and that the enrichment observed within the relatively short and focused Splitter predicted peak regions reflects this observation. Another possible explanation is that the known TH-regulated genes are not, in fact, mediated by direct binding of TR at a TRE (i.e., TR may be interacting with non-TRE sequences).

Considering that TR tends to operate as a dimer, a novel dual-threshold model was developed to scan genomic sequences for putative TREs. Analysis of classification performance estimated the true positive rate of the dual-threshold model to be 0.73 and the false positive rate to be 0.2. Applying this model we identified 85 novel putative TREs in the developing cerebellum.

Overall, we have established a novel pipeline for the identification of TREs and provide a list of candidate binding sites that may be critical for normal TR-driven neurodevelopment. Future research will include wet-laboratory binding site validation, and examination of the other possible binding mechanisms that may be responsible for TR-binding regions not found to contain a putative TRE.

## Methods

### Dataset information

This study was designed using 2 groups of 5 mice each; sacrificed either on post natal day 4 (PND4) or on PND15. ChIP was conducted using an anti-TR antibody and was analyzed alongside total input control DNA. The 10 sample ChIP-chip experiment was conducted on individual Agilent 44k custom tiled promoter region microarrays (Agilent Technologies, Mississauga, ON, Canada) containing 5000 gene promoter regions, with 60 nucleotide long probes mapping the -8 to + 2 kb region of the transcription start site. Probes corresponded to genomic locations every 200 bp on average. All details about the laboratory protocol used to conduct the ChIP-chip experiment and preliminary findings have been described previously [11].

### ChIP-chip analysis

The probe mapping was first updated for the TRE ChIP-chip dataset. Probe chromosomal locations were updated to the (NCBI 37 / MM9) build using the liftOver (http://genome.ucsc.edu/cgi-bin/hgLiftOver) utility provided by the UCSC genome browser.

Popular gene expression normalization methods (Quantile [14] and Lowess [15]) were evaluated for their relevance to ChIP-chip data. The ChIP-chip normalisation method described by Song *et al.*[17] was also considered, but our data did not exhibit the strong bimodal probe intensity distribution seen by Song and the method was therefore not applied. We ultimately used a linear model proposed by Potter *et al.*[18] which corrects for two sources of bias within a single normalization model. The model, which was originally designed for methylation arrays, was modified to also account for the quadratic effect of probe composition. Equation 1 below shows the modified version of the normalization equation presented by Potter *et al.* with its respective parameters described below. The additional quadratic term to account for the nonlinear relationship between probe composition and probe signal intensity is included in the first term of Equation 1 (labelled as ‘A’).

Equation 1: Quadratic model for ChIP-chip normalization derived from Potter *et al*. [15].

The parameters of Equation 1 are defined as follow; *P*_*d*_ is the expected baseline log transformed probe value for d = {Red, Green}, *l* is the number of nucleotides in the probe, i.e. its length, α_0_ is the mean baseline signal across the array_,_ β_j_ is the coefficient for the contribution of base j, for linear and quadratic considerations. The *n*^*’*^_*j*_ parameter is the abundance of nucleotide *j* within the probe divided by *l*, *n*^*’*^_*2j*_ is the abundance of nucleotide *j* in the probe squared divided by *l*, *s*^*’*^_*j*_ is sum of the position of all bases of type *j* within the sequence of the probe divided by *l*, *s*^*’*^_*2j*_ is the sum of the square of the position of base *j* with the sequence of the probe divided by *l*, I is an indicator binary function and δ is the global dye effect.

Splitter [12] was used for ChIP-chip peak identification using the standard deviation as the cut-off parameter to select probes from intensity histogram. The parameter MINRUN and GAPMAX were set respectively at 2 and 200 for the Whitehead dataset [13]. The optimization of the SD parameter was conducted over a range of SDs from 2.25 to 2.75 (increment of 0.05) for every possible combination of these 2 groups: {modified Potter, none} {Splitter} on the Whitehead Agilent dataset from Johnson *et al*[13]*.* The optimal SD parameter value was selected in terms of highest true positive rate and lowest false positive, i.e. the closest coordinal point to the top left corner of Figure 3. Splitter with no normalization was applied to the TRE ChIP-chip dataset with a SD of 2.35, MINRUN of 2 and GAPMAX of 488. ChIP-chip validation of 28 predicted peaks was conducted following the protocols described in Dong *et al*[11].

### Presence of TRE hexamers in genomic DNA

A position weight matrix represents a sequence motif characteristic of a collection of related DNA sequences. In this study, the method of Wasserman *et al.*[40] is used to create all PWM models. Given a PWM, a DNA scanning model then scores the similarity between an input DNA sequence and the sequence motif encoded by the PWM. DR4, IR0, and ER6 TREs were gathered from the literature (see Table 2) and were split into individual hexamers to create a list of half sites. A PWM was compiled from this list of known half sites. This model was used to scan six classes of promoter sequences for half sites using a fixed threshold of 6 for the hexamer cut-off. The six classes of promoter sequences were created as follows: sequence permutations of 50 promoter regions randomly sampled from UCSC Mouse Genome Assembly (MM9) [41] (class a), these same sequences without permutation (class b), promoter regions of genes observed through gene expression study to be controlled by TH [42] (class c) and estrogen [43] (class d), peak regions identified by Splitter (class e), and the subset of these regions validated by ChIP-PCR.

The DNA sequences from Figure 6 were plotted using the same PWM model counting the number of half sites over a sliding widow size of 250bps. The genes used for this scan are (Nr4a1 - NM_010444, Klf9 - NM_010638, Abcd2 - NM_011994 and Cyp7a1 - NM_007824).

### TRE motif finding using a dual-threshold PWM model

Using the list of known half sites (see Table 2), PWMs were compiled from the hexamers using a leave one out (LOO) test protocol, evaluating each half site independently of PWM training, to plot Figure 7. The bimodal distribution suggested a dual-threshold PWM model may be applicable. A bootstrapping test was conducted to test the null hypothesis that the min and max score in each pair of half sites was, in fact, drawn from the same distribution. We first calculated the average difference, d^*^, between the two half site PWM scores for the set of all known mouse TREs. We then computed the distribution of *d* expected under the null hypothesis by applying bootstrapping with 1000 bootstrap samples (pairs of PWM scores drawn from all scores, with replacement). By comparing our observed value of d^*^ relative to the distribution of *d*, we are unable to reject the null hypothesis (p > 0.01). An identical test was performed on TREs from Human, Rat, Mouse, and Chicken reported in Williams [34] with similar results. We recommend that such tests be repeated when more TREs are positively identified and a larger sample of PWM score pairs becomes available.

## Competing interests

The authors declare that they have no competing interests.

## Authors’ contributions

RG conducted the bioinformatics research and drafted the manuscript. HD performed the ChIP-chip and the RT-PCR. CY, JG and RG conceived the project idea. CY and JG supervised the project, provided guidance on the analysis, and critically revised the manuscript. MW provided expert advice on biology, research direction and critically revised the manuscript.

## Supplementary Material

Additional file 1 Histogram of Distribution of Total Input Probes for the Dong Dataset. Click here for file

Additional file 2 **List of Peaks Identified by Splitter in the Dong PND 4 Dataset.** Additional file 2 shows the peaks found by applying the strategy elaborated in this paper to the ChIP-chip data from PND4. The file includes the following columns: the chromosome of the peak, the starting location of the peak, the corrected ending location of the peak, the number of probes in a peak, the gap value for the peak (highest distance between probes of a peak), the median signal intensity across probes within the peak, the ChIP-PCR validation results (NV = not validated, TP = true positive, FP = false positive; see section “Analysis of TR ChIP-chip Data”), the location of the peak with respect to the closest mRNA mapped by the UCSC genome browser on MM9 genome build (e = exon, i = intron, o = outside), the ID of the mRNA mapped, the mRNA symbol, the distance from the closest RNA for outside peaks, and the number of references relating the mRNA symbol to the terms “thyroid hormone” on Pubmed. The microarray data can be obtained at MIAMExpress http://www.ebi.ac.uk/arrayexpress/; accession number E-MEXP-180. Click here for file

Additional file 3 **List of Peaks Identified by Splitter in the Dong PND 15 Dataset.** Additional file 3 shows the peaks found by applying the strategy elaborated in this paper to the ChIP-chip data from PND15. The file includes the following columns; the chromosome of the peak, the starting location of the peak, the corrected ending location of the peak, the number of probes in a peak, the gap value for the peak (highest distance between probes of a peak), the median signal intensity across probes in the peak, the ChIP-PCR validation results (NV = not validated, TP = true positive, FP =false positive; see section “Analysis of TR ChIP-chip Data”), the location of the peak with respect to the closest mRNA mapped by the UCSC genome browser on MM9 genome build (e = exon, i = intron, o = outside), the ID of the mRNA mapped, the mRNA symbol, the distance from the closest RNA for outside peaks, and the number of references relating the mRNA symbol to the terms “thyroid hormone” on Pubmed. The microarray data can be obtained at MIAMExpress http://www.ebi.ac.uk/arrayexpress/. Click here for file

Additional file 4 **List of peaks appearing in both PND4 and PND15 and overlapping with Dong et al [**[11]**].** Click here for file

Additional file 5 **Half site density (y-axis) along the length of the promoter region sequence (x-axis) with the actual TRE location highlighted using an arrow for the eight remaining mouse TH-regulated genes from Table** 2**that were not plotted in Figure** 6**.** Click here for file

Additional file 6 **Putative TREs in Splitter Peaks for PND4.** The table below show the results of our novel dual-threshold PWM scanning approach for PND4. Columns are described as the peakID which is the corresponding identifier to the peakID identifier for PND4 in Additional file 1, strand on which the TRE is located, the start position with respect to the location “start” in Additional file 1, the end position with respect to the location “start” in Additional file 1, first half site, second half site, the PWM score of the first half site, the PWM score of the second half site, and the type of TRE {DR4=Direct repeat with a spacer of 4 nucleotides, IR0= Inverted repeat with no spacer, ER6 = Everted palindrome with a spacer of 6 nucleotides}. Click here for file

Additional file 7 **Putative TREs in Splitter Peaks for PND15.** The table below show the results of the known TRE motif scanning for PND15.Columns are described as the peakID which is the corresponding identifier to the peakID identifier for PND15 in Additional file 2, strand on which the TRE is located, the start position with respect to the location “start” in Additional file 2, the end position with respect to the location “start” in Additional file 2, first half site, second half site, the PWM score of the first half site, the PWM score of the second half site, and the type of TRE {DR4=Direct repeat with a spacer of 4 nucleotides, IR0= Inverted repeat with no spacer, ER6 = Everted palindrome with a spacer of 6 nucleotides}. Click here for file

Additional file 8 **Distribution of putative TRE frequency as determined by our dual-threshold TRE scanning algorithm for the six classes of sequences plotted in Figure** 5**.** Click here for file
