# Supplementary figures and images for: Identification of thyroid hormone receptor binding sites in developing mouse cerebellum
Source: BMC Genomics. 2013 May 23;14:341. doi: 10.1186/1471-2164-14-341 (PMC3716714; doi:10.1186/1471-2164-14-341)

## Slide 1
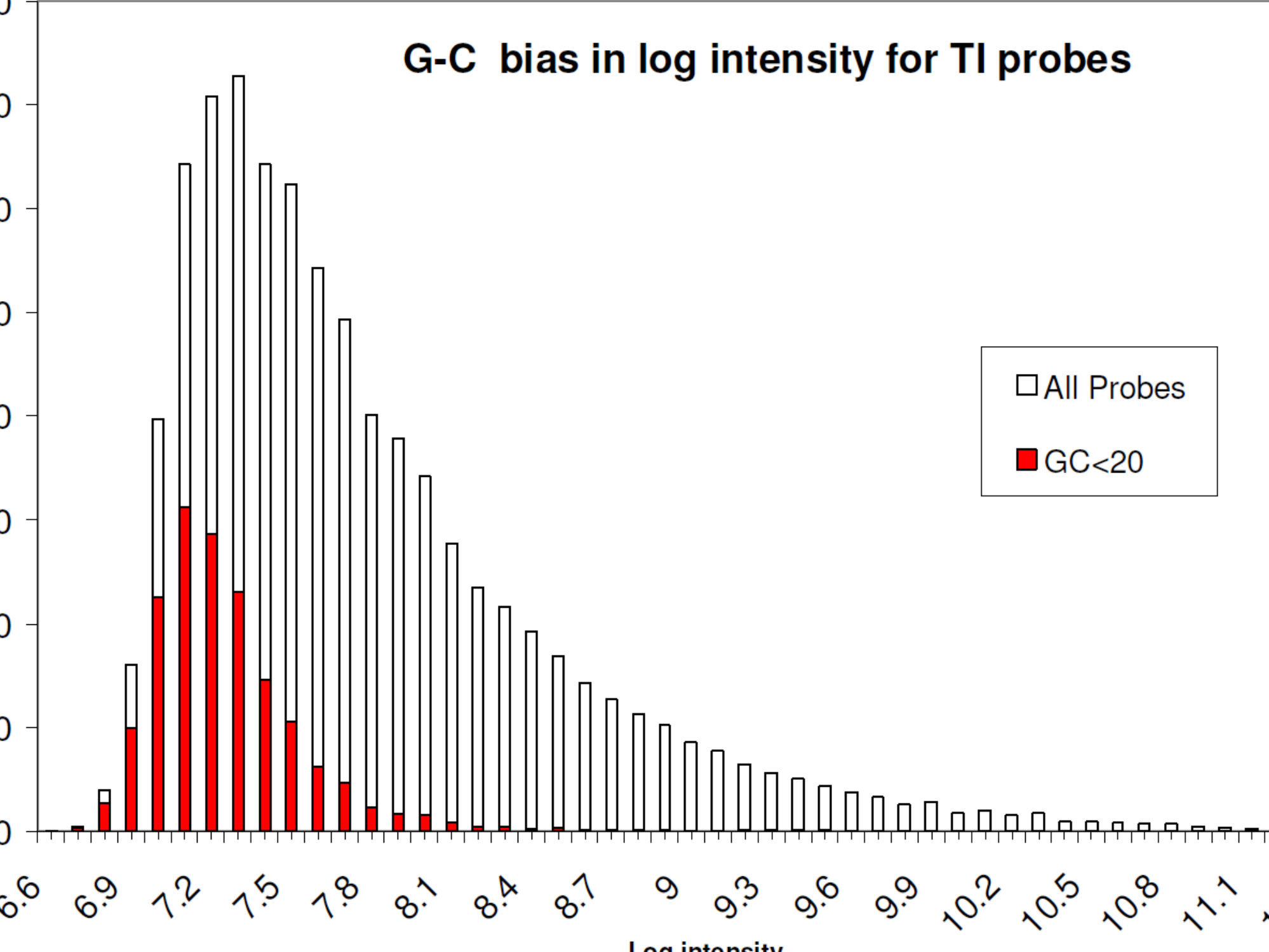

Supplement: Additional file 1 — Histogram of Distribution of Total Input Probes for the Dong Dataset. [file 1471-2164-14-341-S1.ppt]

## Slide 1
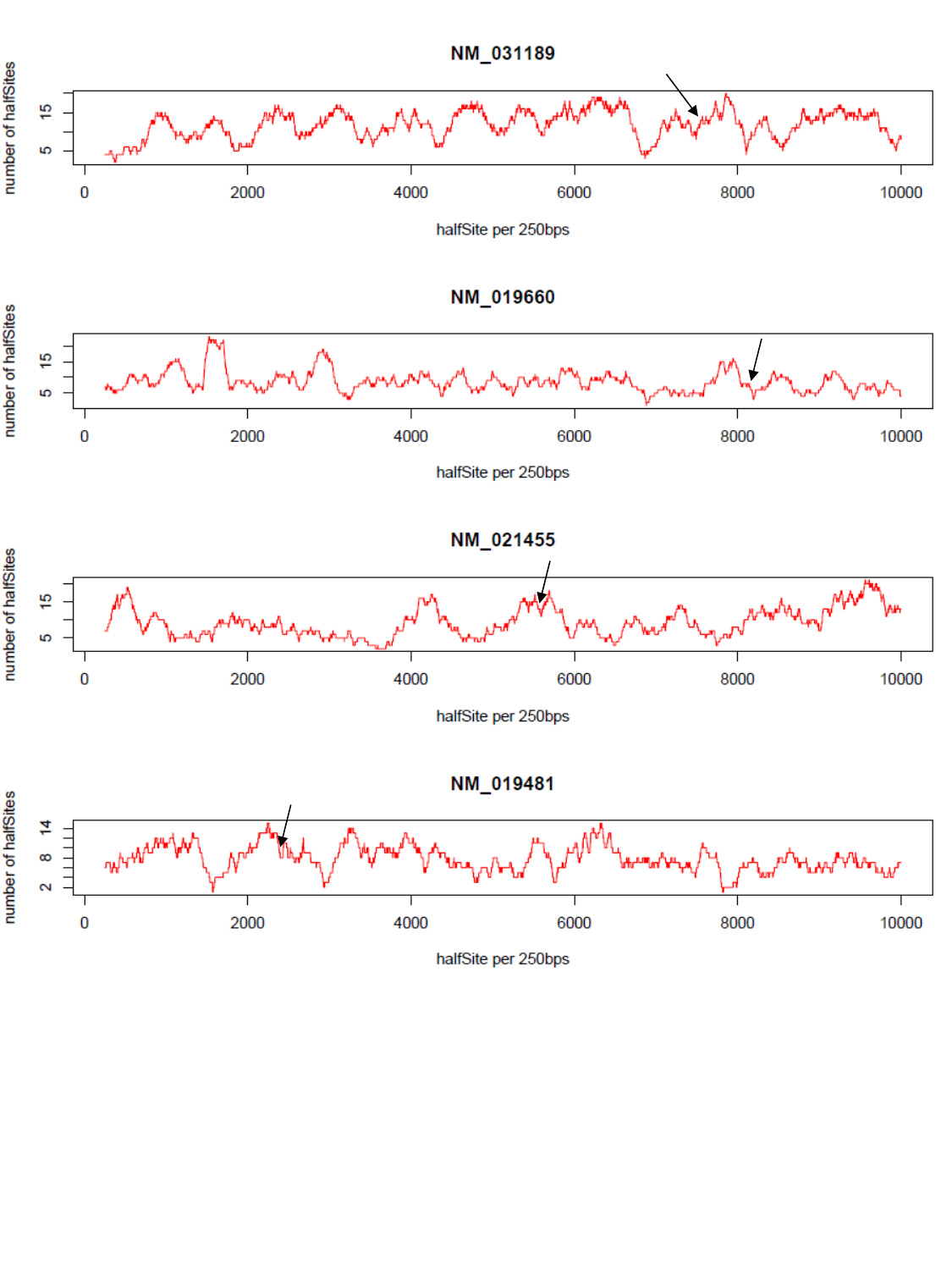

## Slide 2
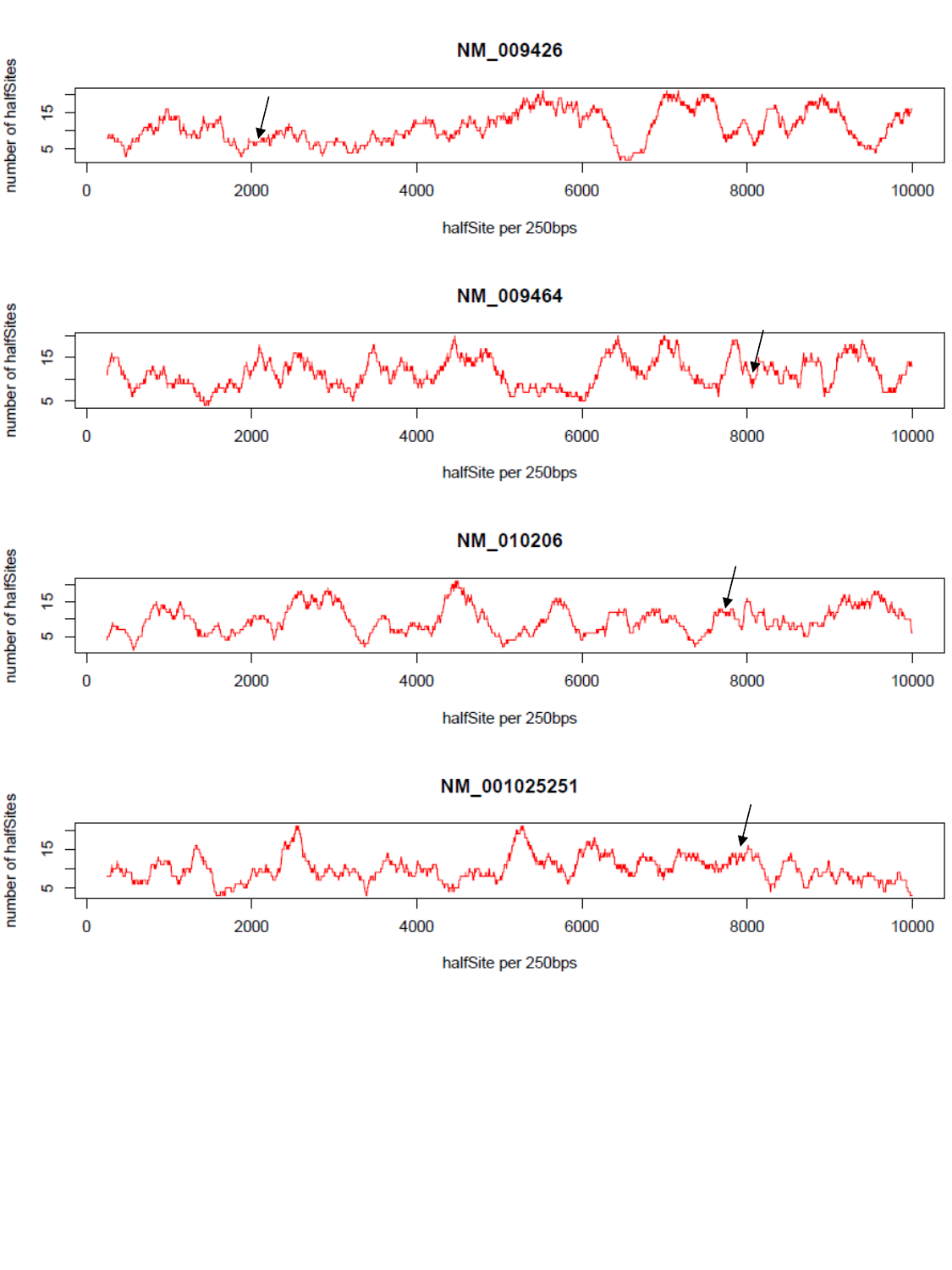

Supplement: Additional file 5 — Half site density (y-axis) along the length of the promoter region sequence (x-axis) with the actual TRE location highlighted using an arrow for the eight remaining mouse TH-regulated genes from Table 2that were not plotted in Figure 6. [file 1471-2164-14-341-S5.ppt]

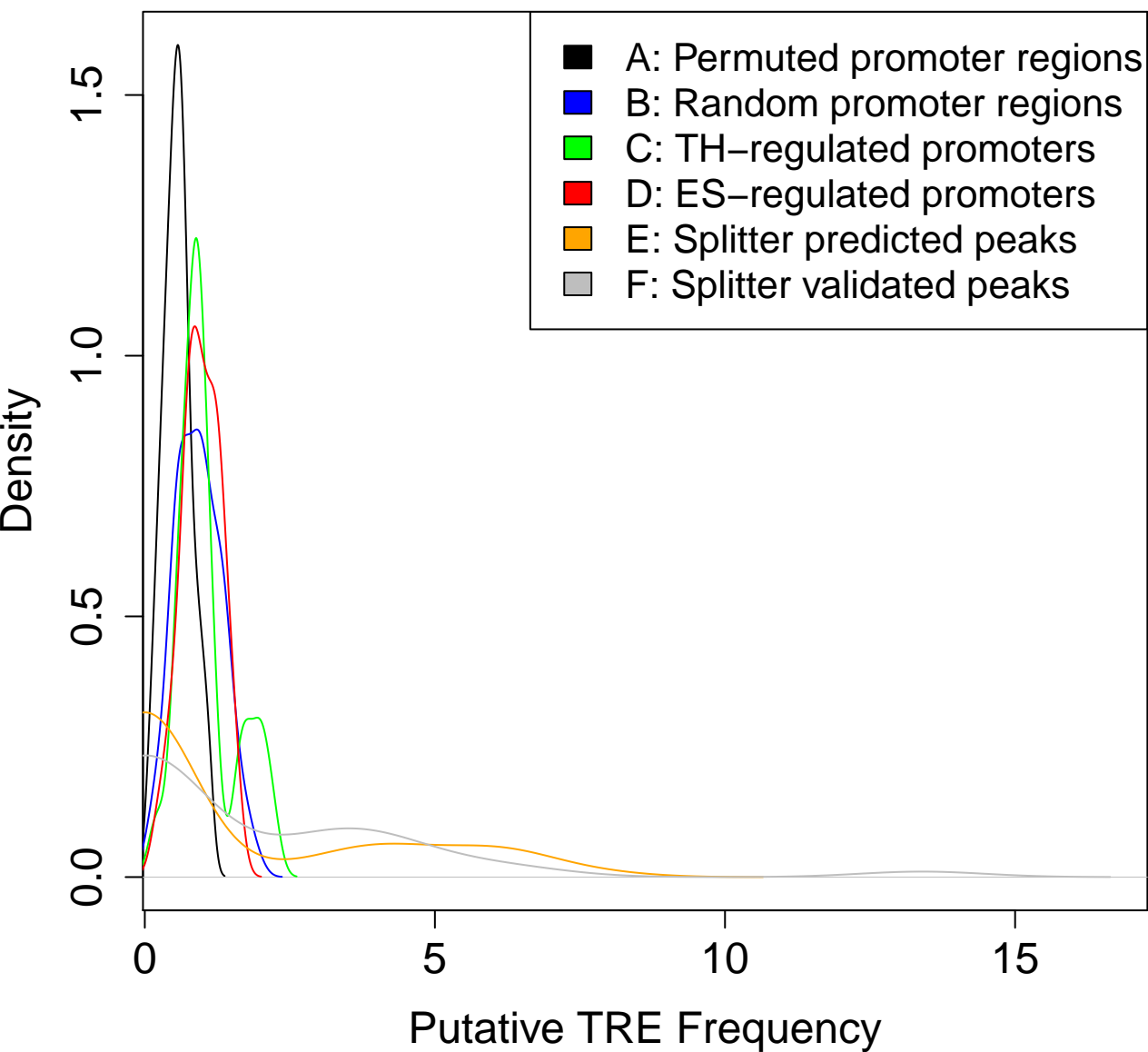

Supplement: Additional file 8 — Distribution of putative TRE frequency as determined by our dual-threshold TRE scanning algorithm for the six classes of sequences plotted in Figure 5. [file 1471-2164-14-341-S8.pdf]
